# 

**Published:** 1898-12

**Authors:** 


### MARRIAGE.

Dr. T. W. Scott, formerly of Nashville, but more recently of Clarksville, Tennessee, was married November 5, 1898, to Miss Annie Oglesby, of the latter city. The ceremony was performed by the Rev. R. A. Webb. The many friends of Dr. Scott who had the pleasure of his acquaintance at Nashville in September, 1897, will wish him and his bride the utmost happiness and prosperity.

### PERSONALS.

Dr. F. D. Markham, of Port Leyden, N. Y., is associated with his father in the management of the Fair View Stock Farm, where he also maintains hospital accommodations for his patients.

Dr. C. S. McKenna, of Washington, Pa., has disposed of his practice to Dr. D. E. Kimmell, of Washington, Pa., and will repair to a drier climate in the hope of regaining his health.

Dr. Harry Walter, of Wilkesbarre, Pa., while on a trip to New York and Philadelphia, was forced to take to his bed in the latter city with a severe attack of the "grippe."

Dr. L. H. Smith, graduate of the Ontario Veterinary College, class of 1897, is a student at the Baltimore Medical College, taking a course in human medicine.

Dr. H. A. Meisner, of Baltimore, Md., was a visitor to Philadelphia on a professional mission in October.

Dr. J. Herbert Snider has removed from Wawanesa to Baldur, Manitoba, and reports prospects as very bright in that territory.

Dr. C. Hervey Bugbee, graduate of the Ontario Veterinary College, has located permanently at Keene, N. H.

Drs. J. M. Phillips and A. Darling acted as veterinarians to the St. Louis horse-show early in November.

Dr. Bernard Gunther, of Brooklyn, N. Y., was a visitor to the JOURNAL office in the latter part of October.

Dr. D. Ryder, of Chambersburg, Pa., is taking a special course of instruction at the Veterinary Department of the University of Pennsylvania.

Dr. Harry Walter, of Wilkesbarre, Pa., was a visitor to the "City of Brotherly Love" and to "Gotham" early in November.

Dr. D. Ryder, of Chambersburg, Pa., and Dr. E. H. Landes, one of Camden's Coroners, recently returned from Tampa, Fla., where they were formerly engaged in the work of stamping out glanders and other infectious and contagious diseases.

Dr. J. P. Turner, of Washington, D. C., Inspector of Dairies for the Health Bureau of Washington, was a visitor to West Chester and Philadelphia in November.

Dr. J. J. Repp, of Philadelphia, recently visited Potter County for the State Livestock Sanitary Board, to investigate an extensive outbreak of verminous bronchitis in some five hundred calves varying in age from five to eight months.

Dr. George Townsend, of New Glasgow, Nova Scotia, holds the position of livestock inspector for that district.

Mr. George H. Metcalfe, of Syracuse, N. Y., late assistant to Dr. J. C. McKenzie, and a third-year student of the New York College of Veterinary Surgeons, committed suicide in a livery stable at Syracuse on October 19, 1898.

Dr. C. J. Alloway, graduate of the class of 1869 at Montreal Veterinary College, was a recent visitor to his alma mater.

Dr. Geo. H. Bailey, State Veterinarian of Maine, has well sustained the position of that Commonwealth on the intelligent management of bovine tuberculosis. Dr. Bailey places New Hampshire in an embarrassing position by pointing out the dangerous methods that State is employing with this scourge.

Mr. R. A. Pearson, of the dairy division of the Department of Agriculture, was a welcome visitor to the November meeting of the Keystone Veterinary Medical Association, and spoke warm words of encouragement of the Society's work in the bettering of the milk-supply of Philadelphia and vicinity.

Dr. L. A. Nutting, of Syracuse, N. Y., met with the loss by death of his father in October.

Dr. P. K. Jones, of Pittsburg, Pa., was a visitor to Philadelphia and also to the New York Horse Show in November.

Dr. F. C. Grenside, of New York, was the judge of saddle-horses at the Cleveland horse-show in November.

Dr. J. H. Pear, formerly of Chicago, Ill., has removed to Saugatuck, Mich.

Dr. R. Lindsey Tritton, of Richmond, Va., graduate of the New York College of Veterinary Surgeons, enjoys a large practice in that Southern city. He has a son among the list of students at his alma mater.

Dr. E. M. Massinger, fills the rôle of milk inspector for the town of Phœnixville, Pa.

Dr. J. O. Reed, of Danville, Pa., was a visitor to Philadelphia in November.

### INDEX.

ABBOTT, A. F., D.V.S., 687
Abnormal position of the kidney, 471
Abortion, etiology of epizoötic, 226
Abscess of the sheath of the flexor pedis perforans tendon, 389
Accidental death from morphine, 91
Acorns, enzoötic gastro-enteritis from, 91
Actinomycosis of the udder, 90
Adams, John W., A.B., V.M.D., 51
Address of welcome, New York State Veterinary Medical Society, 694
Aid the Red Cross organizations, 557
Alexander, A. S., M.D.C., V.S , 388
Allen, Frank, D.V.S., 424
American Veterinary College, 336, 577
    commencement exercises, 361
  Veterinary Medical Association, 635
Among Missouri Valley veterinarians, 553
  Montreal graduates, 476
  the graduates of the Ontario Veterinary College, 479
  the profession in Canada, 475
  the profession in Illinois, 400
  the profession in New York State, 331
Among the colleges:
  American Veterinary College, 577
  Grand Rapids Veterinary College, 759
  Indiana Veterinary College, 576
  Iowa State College of Agriculture and Mechanical Arts, Veterinary Department of, 577
  Kansas City Veterinary College, 575, 704, 759
  McKillip Veterinary College, 576, 704
  Ohio State University, Veterinary Department, 759
  Ontario Veterinary College, 704
  Western Veterinary College, 578, 704, 759
Anacker, Dr., 806
Analogy of irian uveitis in man with dry periodic fluxion in the horse, 89
Anæsthetic, new, 86
  a new local, eucaine, 458
Anæsthetics, 753, 816
  local, 630
An ancient hospital, 680
Ancient scales, 546
Anemone memorosa, 804
Animals, feeding, 253
  growth of, 381
  modified milk for, 232
  some of the more prevalent diseases affecting, 6
Annales de Médecine Veterinaire, 90, 91
Another aspirant for veterinary fame, 409
  curiosity, 410
  remarkable letter, 556
Ante-partum vaginal prolapse, 94
Anti-diphtheria serum in asthma, 680
Antidote for strychnine poisoning, lard as, 461
Antipyrin in ophthalmia, 629
  in sunstroke, 680
  reduction in price of, 628
Antiseptic, a new, 180, 628
  ointment, 629
  powder, 548
    for dusting on unhealed wounds, prescription, 324
  surgical dressing, 630
Antiseptics, comparative value of, 463
Antistreptococcus serum, treatment of purpura hemorrhagica with, 46
Antitoxin, tetanus, 815
  treatment of diphtheria, 546
Antivivisection agitation, 711, 785
An unexpected recovery, 473
Aphthæ, or vesicular stomatitis of the horse, 459
Application for insect-bites and stings, 464
  of medicine in the pulverized form to the organs of respiration, 804
Archiv für Wissenschaftliche, 383
Arecolin in colic, 140
Army drift, 494, 560, 768
  legislation, points in regard to, 408
  veterinarians, ophthalmoscopic course for army, 458
Arsenical paste, painless, 814
Arytenoideraphy, new surgical treatment for roaring, 553, 632
Articular rheumatism, Rœntgen rays as a remedy for, 379
Ascites amnion, 164
Asthma, antidiphtheria serum in, 680
Association of Veterinary Faculties of North America, 573
At it again, 273
  Omaha, 557, 637
Attempt to exterminate mice by means of the shrew-mouse bacillus, 36
Austria, the question of raising the official rank of district veterinarians in, 379
Autointoxication in carnivora, 735
A. V. M. A., 760
  officers and committees, 1898–'99, 704
Axis, transverse fracture of the, 93

BACILLI of bubonic plague, 690
Bacillus, shrew-mouse, attempt to exterminate mice by means of the, 36
  toxin of tuberculosis, 628
Bacteria, fossil, 547
Bacteriological stand-point, milk supply from, 215
Baden-Baden, seventh international congress of veterinary surgeons at, 1899, 491, 836
Bandits who make society their prey, 324
Barbadoes aloes not in the market, 631
Barium chloride, 812
Beef, to detect horseflesh from, 627
Bell, Roscoe R., D.V.S., 647
Berlin, discussion of methods for fighting hoof-and-mouth disease in, 322
Berns, George H., V.S., 651
Biart, E. H., V.S., 689

Bignall, L. M., necrology, 636
Bland, Charles, V.S., 265
Bleeding with the hollow needle, 38
Blood serum for subcutaneous intravenous injection, 813
to stop the flow of, 807
Boric acid, solvent action of, 631
Bovette, E., V.S., necrology, 757
Bovine abortion, milk-fever and garget, the prevalence, cause, and treatment, of 172
tuberculosis, 546
Breeders' Gazette, 388
Bristol (England) Medical News, 543
British Medical Journal, 40
Brown, J. E., V.S., 200
Brubaker, F. B., M.D., 545
Bulletin Pharmacy, 464, 465
Bureau of Animal Industry, control work in, 706
Butterfield, J. F., V.S., 263

Cæsarean section, 754
Calculi, preputial and urethral, 161
Calf-lung, extraordinary large number of cysticercus bovis in a, 38
California, control work in, 706
Calomel, carbolic salve containing, 629
Campbell, A. B., V.S., 469
L. D., V.S., 190, 432, 566
Canada, among the profession in, 475
edition, 441
veterinary associations, 475
Canadian livestock inspectors, 483
notes and personals, 486
Canker of both forefeet, 262
or roup, cause and treatment, 451
Cape of Good Hope Agricultural Journal, 461
Carbolic salve containing calomel, 629
Card of "Windy City" specialist, 406
Carnivora, autointoxication in, 735
Case of spiroptera megastoma (rud), 41
Casper, M., 38, 150
Catalogue, University of Pennsylvania, 1897–'98, new publication, 104
Cataract, succus cineraria maritima for, 812
Caterpillars, poisoning by, 627
Cattle diseases, 625
practice, some cases in, 158
tuberculosis in Denmark, proposed law for the extermination of, 534
in Switzerland, regulations against, 37
question of the extermination of, 532
Centralbl. f. Bakt., 89
Centre of veterinary education, 344
Cerebro-spinal meningitis, 87, 630, 797
Cheap practitioners, 521, 749
Chemical orthography, new, 813
Chester County, tetanus in, 256
Chester, Frederick D., bacteriologist, 797
Chicago's latest effort to raise money for use in improving the streets and parks, 112
Chicago Veterinary College, 402
commencement exercises, 435
Society, 60, 117, 189, 404, 422, 566, 829
Chicken-mite, the internal, 523
Clancy, Jos. B., V.S., 829
Clark, J. L., V.S., 449
W. G., M.D.C., 357
Clement, A. W., V.S., 6
Clinical diagnosis of lameness in the horse, new publications, 103
notes, 396, 398, 705
from the McKillip Veterinary College, 395, 474, 552
reports, New York College of Veterinary Surgeons, 328
Veterinary Hospital, University of Pennsylvania, 165, 260
Clitoris, epithelioma of, 161
Colleges of the Central West, 558
Colic, arecolin in, 140
rectal examination in protracted cases of, 689
Colon, rotation or twist of great, 688
twist or rotation of, 468
Colts, lameness in, contribution to the etiology of, 150
Columbia Veterinary College, 337
Commencement exercises:
American Veterinary College, 361
Chicago Veterinary College, 435
Grand Rapids Veterinary College, 360
Harvard University, School of Veterinary Medicine, 510
McGill University, Veterinary Department, 501
McKillip Veterinary College, 435
New York College of Veterinary Surgeons, 359
New York State Veterinary College, 509
Ontario Veterinary College, 503
School of Veterinary Medicine, Harvard University, 510
University of Pennsylvania, Veterinary Department, 507
United States College of Veterinary Surgeons, 361
Commercial plants of Europe, 815
Comparative statistics of cysticercus cellulose in the eye of man and of animals, 748
value of antiseptics, 463
Complicated myocarditis, 686
Conard, M. E., V.M.D., 237
Contagious cerebro-spinal meningitis, 630
of the horse, 87, 797
Contemplated blow at our horse industry, 168
Control work in:
Bureau of Animal Industry, 706
California, 706
Hawaiian Islands, 175
Illinois, 420
Indiana, 343
Kentucky, 706
Maryland, 109
Massachusetts, 105
Minnesota, 53, 180, 343, 411
Missouri, 343
National, 52, 109
New York, 53
Pennsylvania, 53
Constipation, 472
Contribution to the etiology of lameness in colts, 150
to the hygiene of milk, 133
to the question of tuberculin injection in cattle, 807
Convention of National Master-shoers' Protective Association, 40
Cornell University, Veterinary Department of, 336
Correction, 410
Correspondence, 365, 366, 409, 556
interesting, 131
Cornevin, Charles Ernest, necrology, 171
Counter-irritant, 629
Country practice, useful points of value in a, 614
Cow, distressed breathing in, 263
Cows, eversion of the uterus in, 239
failing to breed, and what to do with them, 221
new treatment of parturient paresis of, 619
Creosote, internal administration of, 463
Cruelty to animals, the close relationship existing between the veterinary profession of to-day and organized societies for the prevention of, 21
Cunean tenectomy, 553
Cryptorchidism, which testicle is most frequently displaced in, 96

Cysticercus bovis, extraordinary large number of, in a calf lung, 38
cellulose in the eye of man and of animals, comparative statistics of, 748

Dairy and milk inspection, 25
-cows, feeding corn smut to, 80
Dalrymple, W. H., M R.C.V.S., 21, 95
Dangers of milk, 12
Death from morphine, 91
Dehorning and castration, 365
Dental cysts, 424
Denmark, proposed law for the extermination of cattle tuberculosis in, 534
Department of surgery, 752, 816
Determination of incipient tuberculosis with the aid of Röntgen rays, 457
Deutsche Thierartzliche Wochenschrift, 37, 39, 156, 322
Digitalis, active constituents of, 814
Diodide acetylene, 628
Diphtheria, antitoxin treatment of, 546
Discussion of methods in Berlin for fighting the hoof-and-mouth disease, 322
Diseases affecting animals, some of the more prevalent, 6
ointment for itching skin, 629
wild and cattle, 625
Disinfection of wounds and instruments by a solution of formal in, 323
Distressed breathing in a cow, 263
District of Columbia, Veterinary Medical Association of, 351
Dog mange, scabinol for, 628
morbus maculosus (purpura hemorrhagica), in a, 327
multiple sclerosis in a, 466
Dogs, remedies worth trying in eczematous eruptions of, 99
Donohue, Florince O., 809
Don't hide your light under a bushel, 270
Dried milk, 675
Drugs, potency of crude, 548

Eczema, Lassar's paste for, 630
Eczematous eruptions of dogs, remedies worth trying in, 99
Editorial, 169, 170, 274, 489, 490, 559, 635, 636, 692, 825, 827
Aid the Red Cross Organizations, 557
At it again, 273
At Omaha, 557
American Veterinary Medical Association, 635
Appointment, excellent, 488
Canada edition, 488
Centre of veterinary education, 344
Colleges of the Central West, 558
Contemplated blow at our horse industry, 168
Don't hide your light under a bushel, 270
Empire State edition, 344
Excellent appointment, 488
For the best equipped meat-inspectors, 100
For better municipal meat-inspection, 48
Horse industry, contemplated blow at, 168
Illinois' opportunity, 407
Important action, 755
Inauguration of State editions of the "Journal," 270
In 1899, 755
Journal, inauguration of State editions of, 270
Leads veterinary journalism in America, 168
Losses in the veterinary world, 100
Manitoba the realm of veterinarians, 489
Editorial—
Meat-inspection, for better municipal, 48
Municipal meat-inspection, 48
Ontario's bad example continues, 271
Pennsylvania's annual meeting, 273
Points in regard to army legislation, 409
Programme is on trial, 558
Report of National Stock Growers' Convention, 409
State work well done, 169
They don't want our meat products, 47
Turn on the light, 756
Value of State laws, 47
Veterinary world, losses in, 100
What will the veterinarians of Illinois do? 407
Where in 1899? 691
Will New York lead? 344
Education, veterinary, preparatory, 526
Effect of tuberculin and glycerin extract from tuberculous organs, 321
Ellis, Robert W., D.V.S., 58, 124, 202, 355, 433, 499, 669, 694, 697, 762
Embryo of the horse, development of the, 750
Empire State edition, 344
Veterinary colleges, 336
Empyema of the cavity of the right upper jawbone in the horse, 156
Enzoötic gastro-enteritis from acorns, 91
Epizoötic abortion, etiology of, 226
Epithelioma of clitoris, 161
Erythema scratches, prescriptions for, 324
Etiology of epizoötic abortion, 326
lameness in colts, contribution to the, 150
of nettle-rash in swine, 806
Eucaine, a new local anæsthetic, 458
Europe, veterinary notes from, 592
Eversion of the uterus in cows, 239
Examination for soundness, observations of the face in, 190
eye of the horse subjected to criticism in, 423
Excessive œdema of the head, 94
Extracts from foreign journals (German):
Anemone memorosa, 804
Attempt to exterminate mice by means of the shrew-mouse bacillus, 36
Bleeding with the hollow needle, 38
Blood, to stop the flow of, 807
Calf lung, extraordinary large number of cysticercus bovis in a, 38
Cattle tuberculosis, proposed law for lhe extermination of, 534
in Switzerland, regulations against,37
question of the extermination of, 532
Cerebro-spinal meningitis, contagious, 87
Cheap practitioners, 531, 749
Colts, contribution to the etiology of lameness in, 150
Contagious cerebro-spinal meningitis of the horse, 87
Contribution to the etiology of lameness in colts, 150
to the question of tuberculin injection in cattle, 807
Cysticercus bovis, extraordinary large number cf in a calf lung, 38
cellulosæ in the eye of man and of animals, comparative statistics of, 748
Discussion of methods in Berlin for fighting the hoof-and-mouth disease, 322
Denmark, proposed law for the extermination of cattle tuberculosis in, 534
Determination of incipient tuberculosis with the aid of Röntgen rays, 457
Development of the embryo of the horse, 740
Effect of tuberculin and of glycerin extract from tuberculous organs, 321

Extracts from foreign journals (German)—
Embryo of the horse, development of the, 750
Empyema of the cavity of the right upper jawbone in the horse, 156
Eucaine, a new local anæsthetic, 458
Extirpation of splenic fever by agricultural regulations, 39
Growth of animals, 381
Hoof-and-mouth disease, discussion of methods in Berlin for fighting the, 322
infection of man with the, 805
Horse, contagious cerebro-spinal meningitis of the, 87
development of the embryo of the, 750
empyema of the cavity of the right upper jawbone in the, 156
spontaneous recovery from a chronic prolapsus penis in a, 380
Macroscopically visible trichina, 322
Mice, attempts to exterminate by means of the shrew-mouse bacillus, 36
New tattooing apparatus, 381
Needle, bleeding with the hollow, 38
Ophthalmoscopic course for army veterinarians, 458
Organo-therapeutics, 751
Organs of respiration, application of medicine in pulverized form to the, 804
Proposed law for the extermination of cattle tuberculosis in Denmark, 534
Physiological and clinical investigation of a white dog with blue eyes, 533
Question of the extermination of cattle tuberculosis, 532
of raising official rank of district veterinarians in Austria, 379
Regulations against cattle tuberculosis in Switzerland, 37
Röntgen rays as a remedy for articular rheumatism, 379
determination of incipient tuberculosis with the aid of, 457
Sewing of wounds, 323
Splenic fever, extirpation of, by agricultural regulations, 39
Spontaneous recovery from a chronic prolapsus penis in the horse, 380
Statistics regarding tuberculosis in the Prussian slaughter-houses, 534
of cysticercus cellulosæ in the eye of man and of animals, comparative, 748
relative to the prevalence of tuberculosis, 676
Tenacity of the aptha contagion, 805
Test of fresh milk, and the impurities contained therein, 750
Tetanus therapeutics, 804
Therapeutics of wounds in the joints and ligaments, 322
Tuberculin and glycerin extract, 321
Tuberculosis, cattle, question of the extirmination of, 532
in the Prussian slaughter-houses, statistics regarding, 534
some statistics relative to the prevalence of, 676
Trichina, macroscopically visible, 322
Trichosis on the tongue of cattle, 806
Veterinarians in Austria, question of raising the official rank of district, 379
ophthalmoscopic course for army, 458
Wounds in the joints and ligaments, therapeutics of, 322
Extracts from foreign journals (French):
Accidental death from morphine, 91
Acorns, enzoötic gastro-enteritis from, 91
Analogy of irian uveitis in man, with dry periodic fluxion in the horse, 89
Extracts from foreign journals (French)—
Disinfection of wounds and instruments by a solution of formalin, 323
Enzoötic gastro-enteritis from acorns, 91
Erythema scratches, prescription for, 324
Formalin, disinfection of wounds and instruments by a solution of, 323
Glanders, researches as to the value of mallein as a diagnostic agent in, 382
Monodactyle pigs, 91
Morphine, accidental death from, 91
Pigs, monodactyle, 91
Prescriptions, 324
Pruritus of the skin and erythema where there is intense itching, prescription for, 324
Researches as to the value of mallein as a diagnostic agent in glanders, 382
Udder, actinomycosis of the, 90
Uterus, to counteract premature spasmodic contraction of the, prescription for, 324
Unguentum eucaine for erythema, inflammation of the mucous membrane and any painful swollen surface, prescription for, 324
Verminous typhlitis, 91
Wounds, antiseptic powder for dusting on unhealed, prescription for, 324
unguentum sozoidol for, prescription for, 324
Extraordinary large number of cysticercus bovis in a calf lung, 38
Excellent appointment, 488
Excessive œdema of the head, 94
Exciting cause of hydrophobia, 631
Exercises, 510
Extermination of cattle tuberculosis, question of, 532
in Denmark, proposed law for the, 534
Extirpation of splenic fever by agricultural regulations, 39
of the parotid gland, 166
Eyes of the horse subjected to criticism in examination for soundness, 423

FARMERS' Review, 388
Fatty degeneration of a kidney in a cat, 261
Feeding animals, 253
corn smut to dairy cows, 80
Felton, H. B., V.M.D., 245, 283
Femoro-tibial articulation, two cases of lameness in the region of the, 97
Fenimore, H. D., D.V.S., 625
Fever, splenic, extirpation of by agricultural regulations, 39
Fisher, C. W., 16, 60, 117, 202
Flexor pedis perforans tendon, abscess of the sheath of the, 389
Fly-blister, veterinary, 680
Formaldehyde, 549
disinfection without apparatus, 679
Formalin, disinfection of wounds and instruments by a solution of, 322
For pure food, 383
Fossil bacteria, 547
Fracture of ilium, 328
of penis, 160
amputation, recovery, 399
transverse, of the axis, 93
Frame, David P., M.D.C., necrology, 172
Further contribution to the hygiene of milk, 133

GASTRO-ENTERITIS, enzoötic, from acorns, 91
Genessee Valley Veterinary Medical Association, 60, 339, 569
Giffen, Thomas M.R.C.V.S., necrology, 757

Gill, H. D., V.S , 295
Glanders, farcy, 411
researches as to the value of mallein as a diagnostic agent in, 382
Gleanings, 35, 56, 111, 115, 259
Glass, Alexander, V.S., 89, 323
Goff, W. M., 838
Graham, O. W., V.S., 472
Grandclement, Dr., 90
Grand Rapids Veterinary College, 759
commencement exercises, 360
Great trees, 627
Grenside, Frederic, V S., 793
Gribble, Wm. H., D.V.S., 207
Griiner, C. F., M.D.C., 566
Growth of animals, 381

Hæmaturia from umbilical vein, 472
Hall's solution of strychnine, 631
Harger, S. J. J., V.M.D., 96, 140, 165, 226, 249, 260, 422, 471, 735, 821
Harrison, R. H., D V.S . 158
Harshberger, J. W., Ph.D., 143
Harvard University, School of Veterinary Medicine, commencement exercises, 510
Hawaiian Islands, control work in, 175
Hay, L., V.S., 572
Head, excessive œdema of the, 94
Healing salves, 464
Heart, wounds of, not always fatal, 803
Heath, W. H., M.D., 315
Heck, W. A., V.S., 359, 763
Heitzman, Charles W., M.D.C., 666
Helmer, Jacob, D V.S., 703
Henderson, James, M.R.C.V.S., 61, 122
Here and there, 112
Hilton, George, 441
Hodgkin's disease or lymphadenoma, 43
Hog-cholera, State control of, 601
Hogs, swill-fed, powdered soap as a cause of death among, 306
Hoffman, F. F., V.S., 754
Hollingsworth, W. G., D.V.S., 46, 329
Hollow needle, bleeding with, 38
Hoof-and-mouth disease, infection of man with the, 805
discussion of methods in Berlin for fighting the, 322
ointment, 680
Hoofs, toe-divided shoes for contracted, 377
Hoskins, J. Preston, Ph.D., 36, 87, 150, 321, 379, 457, 531, 748, 804
W. Horace, D.V.S., 43, 422, 494, 680
Hospital, an ancient, 680
Horse, analogy of irian uveitis in man, with dry periodic fluxion in the, 89
aphthæ or vesicular stomatitis in the, 459
contagious cerebro-spinal meningitis of the, 87, 797
development of the embryo of the, 750
empyema of the cavity of the right upper jawbone in the, 156
eyes of, subjected to criticism in examination for soundness, 423
industry, contemplated blow at our, 168
in motion, review of, 346
spontaneous recovery from a chronic prolapsus penis in the, 380
Horseflesh, to detect from beef, 627
Horses, method of generating and administering medicated steam to, 528
mouth, 793
nail wounds of the feet of, 647
stomach staggers in, 633
How one of our editors is regarded by the lay press, 543
Hughes, Joseph, V.S , 428
Hutcheon, D., Colonial V.S., 459
Hunter, A. L., V.S., necrology, 636
Hygiene and demography, ninth international congress of, 127
of milk, a further contribution to the, 133
Hydrophobia, exciting cause of, 631
Hypodermic use, quinine for, 628
Hydroquinine, 815

Illinois, among the profession in, 400
associations, 405
control work in, 420
opportunity, 407
State edition, 369
laws, 404
Veterinary Medical Association, 404
Ilium, fracture of, 328
Immunity, 515
Impaction of the rectum, 471
Important action, 755
meeting, 101
In 1899, 755
Inauguration of the State editions of the "Journal," 270
Indiana, control work in, 343
Veterinary College, changes in, 576
Infection of man with the hoof-and-mouth disease, 805
Inspection, slaughter-house, 720
Inspectors, Canadian livestock, 483
Instruments, corrosion of surgical, 815
Interesting correspondence, 131
Internal administration of creosote, 463
chicken-mite (cytodites nudis vizoll), 523
Iowa State College of Agricultural and Mechanical Arts, Veterinary Department of, 577
State Veterinary Medical Association, 197
Veterinary Society, 355
Irian uveitis in man, with dry periodic fluxion in the horse, analogy of, 89
Is the actual cautery beneficial in ringbone or not? 265

Jacob, M., 62, 125, 208, 293
Jackson, Roy E., V.S., 686
James, H. E., V S., 471, 473
James and Perley, veterinary surgeons, 473
Jersey Breeders' Association, Pennsylvania, 292
Jobson, George B., V.S., 221, 703
Joints and ligaments, therapeutics of wounds in, 322
Jopling, William, 205
Journal de Prac., 324
inauguration of State editions of, 270
special to the U. S. V. M. A., Omaha, 1898, 492
Journals, extracts from foreign, 36, 87, 150, 321, 379, 457, 531, 676

Kansas City Veterinary College, 575, 704, 758
Keystone Veterinary Medical Association, 63, 125, 205, 433, 574, 834
Kidney, abnormal position of, 471
in a cat, fatty degeneration of, 261
Keely, H. P., V.M.D., 239
Kelly, A. B., D.V.M., 654, 741, 775
Kentucky, control work in, 706
Kooker, W. S., V.S., 282
Koto, P. O., M.D.C., 73
Kinnell, G. N., M.R.C.V.S., 369

Lameness, an obscure case of, 329
in colts, contribution to the etiology, of, 150

Lameness, two cases of, in the region of the femoro-tibial articulation, 97
two interesting forms of, 42
Lard as an antidote for strychnine-poisoning, 461
Lassar's paste for eczema, 630
Law, James, M.R.C.V.S., 695
Leads veterinary journalism in America, 168
Legislation:
New York, 364
Pennsylvania, 54, 100
Leisure, use of, 673
Lellman, Wilfred, D.V.M., 327, 466
Leuckart, Geheimrat Rudolf, necrology, 274
Lewis, Henry S., M.D.V., 58, 123, 435
Liniment, liquid oleate of ammonia, 548
Liquid oleate of ammonia liniment, 548
Livestock inspectors, Canadian, 483
Long fast, 92
Losses in the veterinary world, 100
Lymphadenoma, or Hodgkin's disease, 43
Lymphoma, 78
Lydtin, 39, 492

Machan, William, V.S., necrology, 757
Macroscopically visible trichina, 322
Maine Veterinary Medical Association, 760
Mallein, 396
as a diagnostic agent, researches as to the value of, 382
Manitoba, the realm of veterinarians, 489
Veterinary Association, 475, 499
Mare, oöphorectomy in a, 166
Martin, W. J., V.S., 389, 463, 546, 679, 812
Marriages, 172, 345, 410, 490, 756, 827
Marshall, C. J., V.M.D., 232
Maryland, control work in, 109
Massachusetts Alumni Association of McGill Veterinary Department, 478
control work in, 105
Veterinary Association, 58, 123, 434
Mayol, 813
Meat and milk inspectors, relation of veterinarians to the public health as, 295
inspection, methods of, 1
inspectors, for the best equipped, 100
Median neurectomy in ringbone and tendonitis, 260
Mediastinal abscess in a cow simulating traumatic pericarditis, 96
Medical News, 35
Medicine, sanitary or preventive, 666
Meeser, John P., D.V.S., necrology, 757
Meningitis, contagious cerebro-spinal, 630
of the horse, 87
Mereshkowsky, S. S., 36
Merillat, L. A., V.S., 68, 474, 632, 752, 816, 820
Mesenteric artery, rupture of, 687
Meso-neurectomy, 552
Method of generating and administering medicated steam to horses, 528
Methods of meat inspection, 1
Mice, attempts to exterminate, by means of the shrew-mouse bacillus, 36
Michener, J. Curtis, V.S., 253, 264, 286, 823
Michigan State Veterinary Medical Association, 203
Microbe of African cattle plague, 808
of pleuro-pneumonia, 535
Microbes, milk a vehicle for, 39
Milk a vehicle for microbes, 39
fresh, a test of, and the impurities contained therein, 750
dried, 675
hygiene of, a further contribution to the, 133
-inspection, dairy and, 25
some dangers of, 12
-supply from the bacteriological standpoint, 215
Milk supply in cities and villiages, municipal control of, 315
Miller, J. L., V.S., 78
Minnesota, control work in, 53, 343, 411
to the front, a suggestion, 570
State Board of Health, Report of the Veterinary Department for quarter and year ending January 1, 1898, 180
Veterinary Medical Association, 571
Missouri, control work in, 343
Valley edition, 515
Society Proceedings, 572
Veterinary Medical Association, 58, 357, 552, 763
Modified milk for young animals, 232
Monodactyle pigs, 91
Montreal Veterinary Medical Association, 475, 832
Moore, R. C., D.V.S., 146, 528
Moore, Veranus A., B.S., M.D., 306
Morbus maculosus (purpura hæmorrhagica) in a dog, 327
Morphine, accidental death from, 91
Multiple sclerosis in a dog, 466
Municipal control of milk-supply in cities and villages, 315
meat inspection, 48
Myocarditis, complicated, 686
McArthur, P. M., necrology, 410
McGill University, Veterinary Department, 475
commencement exercises, 501
Massachusetts Alumni Association of, 478
McGregor, James, 834
McKillip Veterinary College, 403, 704
changes in, 576
clinical notes from, 395, 474, 552
commencement exercises, 435
McLean, C. Courtney, V.S., 25

Nail wounds of the feet of horses, 648
National and State Dairy Laws, new publications, 758
control work, 52, 109
Master-shoers' Protective Association, 40
Live Stock Association of the United States, 465
Necrology:
Bignell, L. M., D.V.S., 636
Bovette, E., V.S., 757
Cornevin, Charles Ernest, 171
Frame, David P., M.D.C., 172
Giffen, Thomas, M.R.C.V.S., 757
Hunter, A. L., V.S., 636
Leuckart, Geneimrat Rudolph, 274
Machan, William, V.S., 757
Meeser, John P., D.V.S., 757
Turner, Frederick William, D.V.S., 757
Turner, La Forest E., D.V.S., 757
Needle, bleeding with hollow, 38
Nelson, Julius, biologist, 172
Newhard, I. C., V.S., 293
New Publications: 828
Clinical Diagnosis of Lameness in the Horse, Wyman, W. E. A., V.S., 103
Horse in Motion, Stillman, J. D. B., 346
National and State Dairy Laws, R. A. Pearson, B.S., 757
Philadelphia Medical Journal, 52
Prevalence, Cause, and Treatment of Bovine Abortion, Milk Fever, and Garget, Nelson, Julius, Biologist, 127
Text-book on Horse-shoeing, Lungwitz: Adams, John W., A.B., V.M.D., 51
University of Pennsylvania Catalogue, 1897–'98, 104
Veterinary Blue-book, 52
Ophthalmology, Van Mater, George C., M.D., D.V.S., 51
New Jersey Agricultural Experiment Station, Bulletin, No. 127, 172

New inventions, 131, 213
tattooing apparatus, 381
treatment of parturient paresis of cows, 619
York College of Veterinary Surgeons, 336
Commencement Exercises, 359
Clinical Reports, 328
control work in, 53
County Veterinary Medical Association of, 57, 123, 338. 201, 354, 432, 498, 697, 761
German Veterinary Medical Association, 339
legislation in, 364
State, among the profession in, 331
associations, 338
State edition, 295
legislation in, 110
Veterinary College, 337
Commencement Exercises, 509
Medical Society, 338, 572, 574, 694
Association, notes taken at, 696
Niagara and Orleans County Veterinary Medical Association, 338
Ninth International Congress of Hygiene and Demography, 127
Noack, Otto G., V.S., 282
Nolan, L. A., 761, 838
North America, Association of Veterinary Faculties of, 573
Carolina Veterinary Medical Association, 432, 697
Notes from clinic of McKillip Veterinary College, 552
from the University of Pennsylvania Veterinary Department, 268
taken at the annual meeting of the New York State Veterinary Medical Association, 696

OBSCURE case of lameness, 329
Observation of the face in examination for soundness, 190
Œdema, excessive, of the head, 94
Officers and Committees, 1898–'99, A. V. M. A., 704
Pennsylvania State Veterinary Medical Association, 491
Ohio State Board of Veterinary Medical Examiners, 762
Veterinary Medical Association, 207
University, Veterinary Department, 759
Ointment, antiseptic, 629
for itching skin diseases, 629
hoof, 680
Oleate of ammonia liniment, 548
Omaha, 1898, 101
some points about the meeting at, 758
Oneida County Veterinary Medical Association, 200, 339
Ontario's bad example continues, 271
Ontario Veterinary College, 478, 704
among the graduates of, 479
Commencement Exercises, 503
Veterinary Medical Society of, 60, 116, 202, 475, 501, 838
Medical Association, 58, 475
Western, 116, 475
Oöphorectomy in a mare, 166
Open joint, 264, 823
Operation for removal of post-nasal fibroid tumor, 550
Ophthalmia, antipyrine in, 629
Ophthalmoscopic course for army veterinarians, 458
Opium, granulated, for making tincture of, 815
Organo-therapeutics, 751
Orthoform, 547
Osteomyelitis generalis, 469
Osteoporosis, 651
Ott, Dr., 133
Our Canada edition, 488
Ox, traumatic pericarditis of the, 146

PAIGE, JAMES B., D.V.S., 676
Partial amputation of the tongue, 191
Parturient apoplexy, milk-fever, etc., 449
paresis of cows, new treatment of, 619
Pasteurization *versus* purity, 245
Pasteur's work, scope of, 542
Pearson, Leonard, B.S., V.M.D., 1, 56, 592
Peculiarities of the lesions of spavin, 249
Penis, fracture of the, 160
amputation, recovery, 399
Pennsylvania, appeal for greater interest in the U. S. V. M. A., 206
a study of the veterinary surgeon's register of, 31
control work in, 53
edition, 214
-Jersey Breeders' Association, 292
legislation, 54
State Board of Veterinary Medical Examiners, 214
Veterinary Examining Board, 422
Medical Association, 209, 275, 498 573, 574, 698
President's address at, 276
Report of Committee on Intelligence and Education, 278
Report of Committee on Resolutions, 289
Report of Committee on Sanitary Science and police, 285
Semi-annual Meeting of, 698
Veterinary Department, University of, notes from, 268
Pennsylvania's Annual Meeting, 273
Veterinary Medical Society, University of, 61, 124, 293
Pensylvanians, what they are thankful for, 267
Percentage of quinine in the different salts of the alkaloids, 546
Perley and James, Veterinary Surgeons, 473
Peroneus tenotomy, 553
Personals, 64, 128, 210, 294, 346, 437, 510, 578, 706, 772, 839
Peters, A. T., D.V.M., 515
Petty, J. W., V.S., 432, 698
Phar. Post and Phar. Review, 463
Philadelphia Medical Journal, 810
new publication, 52
Times, 768
Phillips, Walker S., V.S., 266
Phipps, W. P., V.M.D., 256
Phonendoscope, the, 92
Physiological and clinical investigation of a white dog with blue eyes, 533
Phyto-bezoars, a review of our knowledge of, 143
Pigs, monodactyle, 91
Plague, bacilli of bubonic, 690
Plaskett, Jos., D.V.S., 836
Pleuro-pneumonia, microbe of, 535
Points, 366, 438, 514, 582, 709, 751
about the meeting at Omaha, 758
in country practice, 617
in regard to army legislation, 408
Poisoning by caterpillars, 627
Polypus of the superior and inferior maxillary sinuses, 261

Poor pussy, 544
Popular Science News, 627
Pote, T. B., D.V.S., 12
Potency of crude drugs, 548
Poultry-food, sunflower seeds as a, 548
Posterior tibial neurectomy, 553
Powder, antiseptic, 548
Powdered soap as a cause of death among swill-fed hogs, 306
Practitioners, cheap, 531
Prairie State Veterinary Colleges, 402
Prescriptions, 324
President's Address, Pennsylvania State Veterinary Medical Association, 276
U. S V.M.A., 583
Prevalence, cause, and treatment of bovine abortion, milk fever, and garget, 172
Prevention of cruelty to animals, the close relationship existing between the veterinary profession of to-day and organized societies for the, 21
Proceedings of National Livestock Association, 465
Programme is on trial, 558
Progress of veterinary science, 441
Prolapse, vaginal, 94
Proposed law for the extermination of cattle-tuberculosis in Denmark, 534
Pruriginous erythema a trophic sequel to plantar-neurectomy, 165
Pruritus of the skin and erythema where there is intense itching. prescription for, 324
Puerperal septicæmia (parturient apoplexy, milk fever, etc.), 449
Pure air, 631
Purity, Pasteurization *versus*, 245
Purpura hæmorrhagica, treatment of with antistreptococcus serum, 46
Pyramidon, 815

QUESTION of extermination of cattle tuberculosis, 532
of raising the official rank of district veterinarians in Austria, 379
Quinine for hypodermatic use, 628
Quitman, E. L., M.D.C,, 391

RABIES, 163
and Texas fever in New York, 41
with a report of two recent outbreaks, 654, 741, 775
Ravenel, M. P., M.D., 215
Researches as to the value of mallein as a diagnostic agent in glanders, 382
Rectal examination in protracted cases of colic, 689
Rectum, impaction of, 471
Recueil de Medecine, 324
Red-cross organization, 557
Register of Pennsylvania, study of the veterinary surgeons', 31
Regulations against cattle-tuberculosis in Switzerland, 37
Relation of veterinarians to the public health as meat- and milk-inspectors, 295
Remedies worth trying in eczematous eruptions of dogs, 99
Removal of blood-stains, 548
Reports of cases, 634
Abnormal position of the kidney, 471
Abscess of the sheath of the flexor pedis perforans tendon, 389
Amputation of penis, 399
Ante-partum vaginal prolapse, 94
Ascites amnion, 164
Axis, transverse fracture of the, 93
Arytenoideraphy, a new surgical treatment for roaring, 553, 632

Reports of cases—
Bacilli of bubonic plague, 690
Cæsarean section, 754
Calculi, preputial and urethral, 161
Canker of both forefeet, 262
Case of spiroptera megastoma (rud), 41
Cat, fatty degeneration of the kidney in a, 261
Cattle-practice, some cases in, 158
Clinical notes, 396
Clinical notes from the McKillip Veterinary College, 395, 472, 552
Clinical remarks on contraction of the sole, 821
Clinical reports, New York College of Veterinary Surgeons, 328
Clinical reports, Veterinary Hospital, University of Pennsylvania, 165, 260
Clitoris, epithelioma of, 161
Colic, rectal examination in protracted cases of, 689
Colon, rotation or twist of great, 688
Colon, twist or rotation of the, 468
Complicated myocarditis, 686
Constipation, hæmaturia from umbilical vein, 472
Cow, distressed breathing in a, 263
Cow, mediastinal abscess in simulating traumatic pericarditis, 96
Cunean tenectomy, 553
Cryptorchidism, which testicle is most frequently displaced in, 96
Distressed breathing in a cow, 263
Dog, morbus maculosus (purpura hæmorrhagica) in a, 327
Dog, multiple sclerosis in a, 466
Epithelioma of clitoris, 161
Excessive œdema of the head, 94
Extirpation of the parotid gland, 166
Fatty degeneration of the kidney in a cat, 261
Flexor pedis perforans tendon, abscess of the sheath of the, 389
Fracture of penis, 160, 399
Hæmaturia from umbilical vein, constipation, 472
Head, excessive œdema of the, 94
Hodgkin's disease, lymphadenoma, or, 43
Horses, stomach-staggers in, 633
Impaction of rectum, 471
Influenza, 398
Interesting forms of lameness, 42
Is the actual cautery beneficial in ringbone or not? 265
Kidney, abnormal position of the, 471
Lameness, two interesting forms of, 42
Lameness in the region of the femoro-tibial articulation, two cases of, 97
Lameness, obscure case of, 329
Lymphadenoma, or Hodgkin's Disease, 43
Mare, oophorectomy in a, 166
Median neurectomy in ringbone and tendonitis, 260
Mediastinal abscess in a cow simulating traumatic pericarditis, 86
Mesenteric artery, rupture of, 687
Meso-neurectomy, 552
Morbus maculosus (purpura hæmorrhagica) in a dog, 327
Multiple sclerosis in a dog, 466
Obscure case of lameness, 329
Oophorectomy in a mare, 166
Open joint, 264, 823
Operation for removal of post-nasal fibroid tumor, 550
Osteomyelitis generalis, 469
Parotid gland, extirpation of the, 166
Penis, fracture of, 160, 399
Peroneus tenotomy, 553
Polypus of the superior and inferior maxillary sinuses, 261
Posterior tibial neurectomy, 553

Reports of cases—
Pruriginous erythema, a trophic sequel to plantar-neurectomy, 165
Rabies, 163
Rectal examination in protracted cases of colic, 689
Rectum, impaction of the, 471
Reports of odd cases, 391
Ringbone, is the actual cautery beneficial in, or not? 265
Roaring, a new surgical treatment for, 632
Rotation or twist of great colon, 688
Rupture of the mesenteric artery, 687
Some cases in cattle-practice, 158
Spiroptera megastoma (rud), 41
Stomach-staggers in horses, 633
Tetanus, 266, 329
Three cases of tumors, with their clinical and histological features, 681
Tracheal stenosis, 474
Transverse fracture of the axis, 93
Treatment of purpura hæmorrhagica with antistreptococcus serum, 46
Tumor, operation for removal of post-nasal fibroid, 550
Tumors, three cases of. with their clinical and histological features, 681
Twist or rotation of the colon, 468
Unexpected recovery, 473
Vaginal prolapse, antepartum, 95
Report of Committee on Sanitary Science and Police, Pennsylvania State Veterinary Medical Association, 285
of the Veterinary Department of the Minnesota State Board of Health for quarter and year ending January 1, 1898, 180, 411
of two recent outbreaks, rabies, 654, 741, 775
Reports of odd cases, 391
Repp, John J., 31
Resolutions of the Ontario Veterinary Medical Association, 501
Review, 172, 346
of our knowledge of phyto-bezoars, 143
Revue Veterinaire, 91, 92
Reynolds, M. H., M.D., D.V.M., 188, 571, 601
Rheumatism, articular, Röntgen rays as a remedy for, 379
Rhoads, W. L., D.V.S., 64, 125, 209, 434
Ridge, W. H., V.S., 207, 281
Ringbone and tendonitis, median neurectomy in, 260
is the actual cautery beneficial in? 265
Roaring, new surgical treatment for, 632
Robb, Robert, V.S., M.D., 526
Robertson, James, M.D.C., 190
Röntgen rays as a remedy for articular rheumatism, 379
determination of incipient tuberculosis with the aid of, 457
Rotation or twist of great colon, 689
Roup or canker, cause and treatment, 451
Rupture of the mesenteric artery, 687
Ryan, J. F., D.V.S., 423

SALINE solutions for burns, 813
Sallade, J. W., V.S., 123
Salley, I. L., D.V.S., 760
Salmon, D. E., D.V.M., 583
Sanitation or preventive medicine, 666
*versus* tuberculin in the eradication of tuberculosis, 369
Scabinol for dog-mange, 628
Schneidemuhl, Prof. Dr., 797
Schwarzkopf, Olof, V.M., 619
Schuylkill Valley Veterinary Medical Association, 122, 293
Science *versus* the art of veterinary surgery, 669
Scope of Pasteur's work, 542
Seen and heard in many places, 764
Selections, 39, 324, 383, 535, 809
Semple, Surgeon-Major, 35
Semi-annual meeting of the Pennsylvania State Veterinary Medical Association, 698
Seventh international congress of veterinary surgeons at Baden-Baden, 491, 836
Sewing of wounds, 323
Shaw, Wm. G., V.M.D., 260
Sihler, C. J., V.S., 550
Simpson, Thomas V., V.S., 468
Sisson, S., V.S., 41
Smith, Clinton D., 80
H. W., V.S., 633
N. B., D.V M., 399
Slaughter-house inspection, 720
Society Proceedings:
Association of Veterinary Faculties of North America, 573, 760
Chicago Veterinary Society, 60, 117, 189, 422, 566, 829
District of Columbia, Veterinary Medical Association of, 60, 351
Genessee Valley Veterinary Medical Association, 60, 569
Hygiene and Dermography, Ninth International Congress of, 127
Iowa State Veterinary Medical Association, 197
Iowa Veterinary Society, 355
Keystone Veterinary Medical Association, 63, 125, 205, 433, 574, 834
Maine Veterinary Medical Association, 760
Manitoba Veterinary Association, 499
Massachusetts Veterinary Association, 58, 123, 434
Michigan State Veterinary Medical Assotion, 203
Minnesota State Veterinary Medical Association, 571
Minnesota to the front, a suggestion, 570
Missouri Valley Society Proceedings, 572
Missouri Valley Veterinary Medical Association, 58, 357, 572, 763
Montreal Veterinary Medical Association, 475, 832
New York County, Veterinary Medical Association of, 123, 157
New York State Veterinary Medical Society, 572, 574, 694
Ninth International Congress of Hygiene and Demography, 127
North Carolina Veterinary Medical Association, 432, 697
Ontario Veterinary College, Veterinary Medical Society, 60, 116, 202, 501, 838
Ontario Veterinary Medical Association, 58
Ohio State Board of Veterinary Medical Examiners, 762
Ohio State Veterinary Medical Association, 207
Oneida County Veterinary Medical Association, 200
Pennsylvania, appeal for greater interest in the U S. V. M. A., 206
-Jersey Breeders' Association, 292
State Veterinary Examining Board, 422
Medical Association, 209, 275, 498, 573, 574, 698
Schuylkill Valley Veterinary Medical Association, 122, 293
Semi-annual meeting of the Pennsylvania State Veterinary Medical Association, 698
Seventh International Congress of Veterinary Surgeons, Baden-Baden, 491, 836
Society news, 127
Tennessee Veterinary Medical Association, 834
Thirty-fifth annual meeting U. S. V M. A., 637

Soctety Proceedings—
U. S. V. M. A., 208, 292, 351, 422, 574, 637
Vermont State Veterinary Medical Association, 834
Veterinary Medical Association of New York County, 57, 123, 201, 354, 433, 498, 697, 761
of the District of Columbia, 351
Society of the University of Pennsylvania, 61, 124, 207, 293, 760, 838
Ontario Veterinary College, 60, 116, 202, 501
Western Ontario Veterinary Medical Association, 116, 475
Wisconsin Society of Veterinary Graduates, 356
Society news, 127
Sole, clinical remarks on the contraction of, 821
Some cases in cattle practice, 158
dangers of milk, 12
features of the Manitoba Association, 500
of the more prevalent diseases affecting animals, 6
statistics relative to the prevalence of tuberculosis, 677
Spavin, peculiarities of the lesions of, 249
Spiroptera megastoma (rud), a case of, 41
Splenic fever, extirpation of, by agricultural regulations, 39
Splints, 428
Spontaneous recovery from a chronic prolapsus penis in a horse, 380
Staphylotomy, 395
State control of hog-cholera, 601
editions of the "Journal," inauguration of, 270
laws, value of, 47
University, Champaign, Illinois, 403
work well done, 169
Statistics regarding tuberculosis in the Prussian slaughter-houses, 534
relative to the prevalence of tuberculosis. 676
Stevenson, H. A., M.D., C.M., 451
Stewart, S., M.D., D.V.M., 209, 720
Stiles, Charles W., 110
Stomach-staggers in horses, 633
Study of the Veterinary Surgeons' Register of Pennsylvania, 31
Strychnine, Hall's solution of, 631
-poisoning, lard as an antidote for, 461
Successful operation on a schirrous cord, with fistula, 329
Sunflower seeds as a poultry food, 548
Sunstroke, antipyrine in, 680
Swine, etiology of nettle-rash in, 806
Switzerland, regulations against cattle tuberculosis in, 37

TALLOW tree, 547
Tattooing apparatus, 381
Tegg, George A., M.R.C.V.S., 60, 570
Tenacity of the aphtha-contagion, 805
Tennessee Veterinary Medical Association, 834
Test of fresh milk and the impurities contained therein, 750
Tetanus, 266, 329
in Chester County, 256
Text-book on horse-shoeing, new publication, 51
The close relationship existing between the veterinary profession of to-day and organized societies for the prevention of cruelty to animals, 21
Therapeutic notes, 463, 546, 628, 679, 803, 812, 815
progress, 680
Therapeutics of wounds in the joints and ligaments, 322
tetanus, 804
Thermo-cautery, and its use in veterinary practice, 73
value and use of the, 529
Thierarzt Centralblatt, 323, 379, 458, 532, 749, 804
They don't want our meat-products, 47
Thompson, Hugh, V.S., 396
Three cases of tumors, with their clinical and histological features, 681
Tincture of iodine, decolorized, 629
To detect horseflesh from beef, 627
To destroy worms in powdered vegetable drugs, 464
Toe-divided shoes for contracted hoofs, 377
Tongue, partial amputation of the, 191
Toxin of tuberculosis bacillus, 628
Transverse fracture of the axis, 93
Traumatic pericarditis of the ox, 146
mediastinal abscess in a cow simulating, 96
Treacy, M. J., M.R.C.V.S., 377
Treatment of parturient paresis of cows, 619
of purpura hæmorrhagica with antistreptococcus serum, 46
Trichina, macroscopically visible, 322
Trichosis on the tongue of cattle, 806
Tuberculin and glycerin extract from tuberculous organs, effect of, 321
Tuberculosis, a new remedy for, 819
bacillus, toxin of, 628
bovine, 546
and its relation to the veterinarian, 16
incipient, determination of, with the aid of Röntgen rays, 457
in the Prussian slaughter-houses, statistics regarding, 534
sanitation versus tuberculin in the eradication of, 369
some statistics relative to the prevalence of, 677
State examination of milk for, 809
Tucker, George P., V.S., 529
Tumor, operation for removal of post-nasal, 550
Tumors, three cases of, with their clinical and histological features, 681
Turner, Frederick Wm., D.V.S., necrology, 757
J. P., V.M.D., 354
La Forest E., D.V.S., necrology, 757
W. D., M.D., 461
Turn on the light, 756
Twist or rotation of the colon, 468
Two cases of lameness in the region of the femoro-tibial articulation, 97
interesting forms of lameness, 42
Typhlitis, verminous, 91

UDDER, actinomycosis of the, 90
Ulcers, phosphoric acid for foul, 630
Umbilical vein, hæmaturia from, 472
United States College of Veterinary Surgeons, Commencement Exercises, 361
Unguentum eucaine for erythema, inflammation of the mucous membrane, and any painful swollen surfaces, prescription for, 324
soziodol for wounds, prescription for, 324
University of Pennsylvania Catalogue, 1897–98, 104
Veterinary Department, Commencement Exercises, 507
Hospital, Clinic Report, 165, 260
Medical Society of, 61, 124, 207, 293, 760
Urticaria, 533
Useful points of value in a country practice, 614

Use of leisure, 673
U. S. V. M. A., 126, 208, 292, 351, 422, 574
"Journal" special to, 492
President's address, 583
Thirty-fourth Annual Meeting, 564, 637
Uterine inertia, 631
Uterus in cows, eversion of the, 239
to counteract premature spasmodic contraction of, prescription for, 324

VAN ES, L., M.D., V.S., 681
Van Mater, George C., M.D., D.V.S., 51
Value and use of the thermo-cautery, 529
of State laws, 47
Vaginal prolapse, ante-partum, 94
Vein, umbilical, hæmaturia from constipation, 472
Virginia Medical Semi-Monthly, 462
Verminous typhilitis, 91
Vermont State Veterinary Medical Association, 834
Veterinarian, tuberculosis and its relation to the, 16
Veterinarians, army, ophthalmoscopic course for, 458
Illinois, what they need, 405
Veterinary Blue-book, new publications, 52
Department, Cornell University, 336
Iowa State College of Agriculture and Mechanical Arts, 577
of McGill University, 475
of University of Pennsylvania, Commencement Exercises, 507
clinical notes, 165, 260
Journal and Annals of Comparative Pathology, 542
fly-blisters, 680
Medical Association of New York County, 57, 123, 201, 338, 354, 433, 498, 697, 761
of the District of Columbia, 351
Society of the University of Pennsylvania, 61, 124, 207, 293, 760, 838
Ontario Veterinary College, 60, 116, 302, 501
notes from Europe, 592
Ophthalmology, new publication, 51
Practice, Thermo-cautery and its Use in, 73
Veterinary preparatory education, 526
science, progress of, 441
Surgeons' Register of Pennsylvania, a Study of, 31
Surgery, Science *versus* the Art of, 669
Viceregal visit, 104

WARNER, G. L., D.V.S., 93
Warts, to remove, 465
Waste of human life, 824
Webber, L. R., V.S., 398
Western Ontario Veterinary Medical Association, 116, 475
Veterinary College, 578, 704, 759
Wilcox, E. V., 523, 625, 711, 785
Wild and cattle diseases, 625
Williamson, A. E., V.S., 688
Will New York lead? 344
Wilson, William M., V.S., 673
Wine of cascara sagrada, 630
Wisconsin Society of Veterinary Graduates, 356
What a New York house has done, 340
What Illinois veterinarians need, 405
What is medicine? 464
What New York veterinarians want, 339
What our dairy cattle inherit, 237
What Pennsylvanians are thankful for, 267
What will the veterinarians of Illinois do? 407
Where in 1899? 691
Which testicle is most frequently displaced in cryptorchidism, 96
Whitbeck, S. S., D.V.M., 614
White, David S., 763
Why cows fail to breed, and what to do with them, 221
Wolf-teeth, a few words anent, 68
-in-the-tail, 385
Worms, A. C., M.D.C., 190
Wounds, antiseptic powder for dusting on unhealed, prescription for, 324
Wounds of the heart not always fatal, 803
powder for, 631
sewing of, 323
unguentum sozoidol for, prescription, 324
Wyman, W. E. A., V.S., 42, 97, 103, 816

ZEITSCHRIFT für Fleisch und Milch Hygiene, 133

